# Directional Liquid Transport on Biomimetic Surface with Wedge-Shaped Pattern: Mechanism, Construction, and Applications

**DOI:** 10.3390/biomimetics10050298

**Published:** 2025-05-08

**Authors:** Qing’an Meng, Junjie Zhou, Jie Pang, Luofeng Wang, Kaicheng Yang, Zhangcan Li, Jiayu Xie

**Affiliations:** College of Aviation Engineering, Civil Aviation Flight University of China, Chengdu 641419, China; 15183240257@163.com (J.Z.); hakydn@126.com (L.W.); kc1730019975@126.com (K.Y.); leo00415@163.com (Z.L.); xiejiayu1983@gmail.com (J.X.)

**Keywords:** biomimetic, directional liquid transport, wedge-shaped pattern surface

## Abstract

Natural organisms have evolved highly sophisticated mechanisms for managing water across a broad range of environmental conditions, from arid to highly humid regions. Among these mechanisms, directional liquid transport (DLT) is particularly noteworthy, as it relies on structural designs that facilitate the spontaneous movement of liquids along predefined pathways without the need for external energy sources. The increasing interest in DLT systems is primarily driven by their potential applications in fields such as microfluidics, water harvesting, and biomedical engineering. The focus on DLT is motivated by its ability to inspire efficient, energy-independent liquid transport technologies, which hold significant promise for both fundamental research and practical applications. Notably, wedge-shaped DLT systems have emerged as a particularly promising area of study due to their advantages in terms of manufacturability, liquid collection efficiency, and scalability—attributes that are essential for industrial deployment. This review seeks to explore natural wedge-based DLT systems, providing an in-depth analysis of their underlying principles and their potential for engineering replication. The discussion includes examples from nature, such as desert beetles and spider silk, and explores the theoretical mechanisms governing these systems, including the role of surface energy gradients and Laplace pressure. Additionally, the review highlights advanced fabrication techniques, such as photolithography and laser micromachining, which are crucial for the development of these systems in practical applications.

## 1. Introduction

Natural organisms have evolved intricate structural and physicochemical integration mechanisms to efficiently manage water in both arid and highly humid environments [1,2,3,4,5,6,7]. These systems demonstrate exceptional adaptability through multi-scale structural optimization. Central to these biological strategies is the hierarchical architecture that transforms nanoscale interfacial phenomena into macroscale functional outcomes—a design principle that has driven significant advances in liquid transport systems. Of particular scientific interest is directional liquid transport (DLT), an energy-independent phenomenon that enables the spontaneous movement of liquids along predefined pathways. DLT holds considerable potential for diverse applications, including microfluidic devices [8,9] biomedical engineering [10], atmospheric water harvesting [11,12], and thermal management systems [13].

The evolutionary refinement of natural DLT systems is exemplified through two distinct structural paradigms. One-dimensional (1D) configurations, such as spider silk, cactus spines, and Sarracenia trichomes, employ conical geometries with hierarchical microgrooves that induce Laplace pressure gradients via curvature-driven capillary forces. In contrast, two-dimensional (2D) systems, such as the elytra of desert beetles and the peristomes of Nepenthes, utilize wettability-patterned surfaces with microcavity arrays to generate continuous surface energy gradients. Recent advances in biomimetic fabrication, particularly through additive manufacturing techniques like high-resolution 3D printing, have made it possible to accurately replicate these hierarchical micro/nanostructures. These breakthroughs provide unprecedented control over transport parameters, such as flow velocity and directional switching, through the integration of intelligent materials.

Emerging research highlights the increasing importance of wedge-patterned planar structures as a distinct category within DLT engineering. In comparison to conventional 1D linear systems and complex 3D architectures, wedge-based geometries offer several advantages in three key areas: (1) enhanced manufacturability through scalable techniques; (2) improved liquid collection efficiency due to the synergistic effects of chemical gradients and Laplace pressure gradients; and (3) compatibility with large-area substrates, which is crucial for industrial applications. Despite considerable experimental progress, a systematic synthesis of transport mechanisms and performance evaluation metrics remains conspicuously absent from the existing literature.

In this review, we present a comprehensive framework for nature-derived wedge-based directional liquid transport (DLT) systems, structured around five key methodological components. First, we explore natural paradigms of directional liquid transport, drawing examples from organisms such as desert beetles, spider silk, cacti, and pitcher plants. Second, we analyze the theoretical mechanisms that govern wedge-shaped DLT systems, with a particular focus on the interplay between surface energy gradients and Laplace pressure gradients induced by asymmetric geometric confinement. Third, we examine the fabrication of wedge-shaped surfaces, highlighting techniques such as photolithography and laser micromachining. Fourth, we survey a range of applications, including water harvesting, microfluidics, energy conversion, wearable sweat management, and sensing technologies. Finally, we synthesize design principles derived from biological systems, discuss ongoing challenges, and propose future research directions, including the development of stimuli-responsive materials, multi-physics integration, and AI-driven manufacturing processes.

## 2. Directional Liquid Transport on Natural Systems

**Figure 1:**
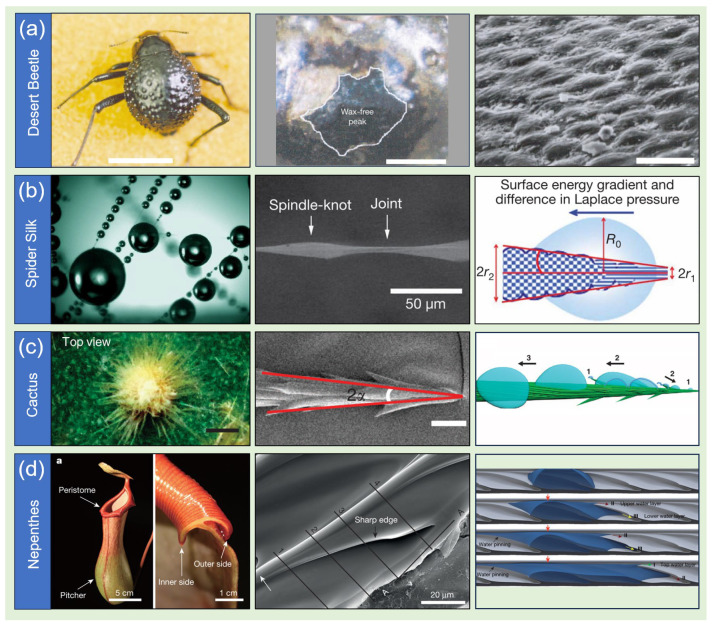
Directional liquid transport on natural systems: (**a**) desert beetle [1]; (**b**) spider silk [2]; (**c**) cactus spine [3]; (**d**) Nepenthes peristome [4].

Taking the desert beetle as a representative example, its elytra surface exhibits non-uniform wettability through the precise arrangement of hydrophobic and hydrophilic domains, thereby forming an active water-harvesting network [1]. The hydrophilic regions serve as “nucleation sites” that preferentially capture fog droplets, while the hydrophobic zones guide droplet migration toward these sites via repulsive interactions. This selective wetting mechanism promotes the rapid coalescence of dispersed droplets until a critical mass is reached. Once gravitational forces surpass the surface tension equilibrium, the droplets roll along predefined pathways, a process that is further facilitated by the beetle’s active postural adjustments. Biomimetic experiments confirm that ordered hydrophobic-hydrophilic composite structures significantly outperform homogeneous surfaces in water collection efficiency, underscoring the critical role of heterogeneous wettability interfaces in enhancing moisture capture (Figure 1a).

Spider silk distinguishes itself through dynamic responsiveness. Upon hydration, the initially loose nanofibril network undergoes structural reorganization, forming periodic spindle-knots interspersed with joints [2]. The spindle-knots, characterized by disordered nanofibrils and increased hydrophilicity, contrast with the joints, which feature aligned fibrils and smoother surfaces. This structural duality generates a continuous energy gradient along the fiber axis, driving the directional migration of microdroplets from low- to high-energy regions. Simultaneously, the conical geometry of the spindle-knots induces Laplace pressure differences, creating dual driving forces—surface energy gradients and pressure differentials—that collectively overcome energy barriers in microscale droplet motion. This cooperative mechanism enables autonomous liquid transport without external intervention, serving as an inspiration for the development of self-driven microfluidic systems (Figure 1b).

The cactus’s fog collection system exemplifies integrated functional modularity. Its spine-like structure consists of three specialized zones: (1) barbed tips that generate pressure gradients through conical geometry, facilitating initial droplet deposition; (2) mid-sections with graded surface roughness that establish energy gradients for directional transport; and (3) basal trichomes that enable rapid absorption via porous hydrophilic structures [3]. This “capture-transport-absorb” tripartite design facilitates counter-gravity water transport, even in the absence of inclination, demonstrating exceptional structural versatility. Biomimetic devices replicating this hierarchical architecture have shown significantly improved stability and efficiency under complex environmental conditions, highlighting the advantages of modular design in addressing challenges related to multi-physics coupling (Figure 1c).

The pitcher plant’s peristome redefines fluid control paradigms through geometric manipulation alone. Its nested microgrooves and duckbill-shaped cavities create multilevel transport channels: sharp cavity edges physically constrain reverse flow via Gibbs pinning, while the graded opening angles of the cavities enhance capillary effects [4]. This architecture sustains unidirectional water transport on superhydrophilic surfaces, effectively addressing the inherent fluid dispersion challenges posed by hydrophilic materials. Notably, the system’s performance is entirely dependent on geometric parameters rather than chemical modifications, presenting novel opportunities for the development of weather-resistant biomimetic materials (Figure 1d).

Collectively, these examples highlight a fundamental principle: natural systems transform basic physical phenomena—such as surface tension, capillarity, and pressure gradients—into robust functional outputs through multi-scale structural coupling. The desert beetle controls droplet coalescence pathways via micrometer-scale wettability heterogeneity; spiders create dynamic energy landscapes through nanoscale fibril reorganization; cacti achieve multifunctional synergy through millimeter-scale spine clusters; and pitcher plants regulate fluid pinning via submicron edge sharpness. Such cross-scale structural cooperation enables biological systems to perform complex functions through geometric precision rather than chemical complexity. Contemporary biomimetic research is evolving from single structural imitations to comprehensive system designs that integrate multi-physical effects. For example, combining the beetle’s wettability heterogeneity with the pitcher plant’s geometric confinement could lead to surfaces that combine high-efficiency water harvesting with anti-fouling properties. Future challenges involve deciphering the implicit “design rules” embedded in biological structures through interdisciplinary approaches and translating these principles into scalable engineering strategies, thus paving the way for innovative technological advances in energy, environmental, and biomedical applications.

## 3. Theoretical Mechanism

**Figure 2:**
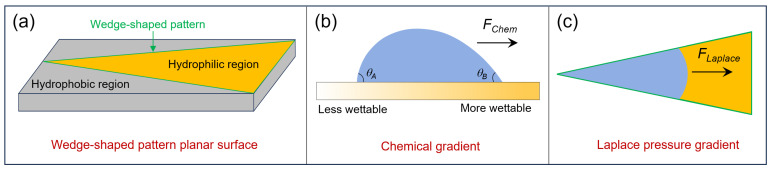
Basic theories about directional transport of a liquid droplet on a wedge-shaped pattern surface. (**a**) The configuration of a wedge-shaped pattern surface; (**b**) directional transport of droplets on a chemical gradient surface; (**c**) directional transport of droplets in a wedge-shaped pattern surface.

The bionic planar wedge-shaped pattern architecture utilizes a hierarchical conical geometry to synergistically combine surface energy gradients with curvature-induced Laplace pressure gradients, enabling precise directional transport and targeted coalescence of microscale water droplets. As illustrated in Figure 2a, the system consists of three key structural components: a hydrophobic exterior that promotes droplet nucleation, wedge-shaped conical patterns featuring a geometrically tapered morphology, and hydrophilic interior zones that facilitate liquid collection.

The dynamics of droplet movement in this system emerge from two interconnected mechanisms. First, the inherent surface energy contrast between the hydrophobic substrate and the hydrophilic patterned regions generates a thermodynamic driving force, which promotes the spontaneous migration of nucleated microdroplets toward the higher-energy hydrophilic zones. In these regions, successive coalescence events lead to the formation of macroscale liquid domains. Second, and critically, the geometrically tapered morphology of the wedge structures introduces a spatially varying Laplace pressure gradient, a curvature-dependent propulsion mechanism that further amplifies directional transport.

Droplets initially nucleate and coalesce within the hydrophobic peripheral regions before displaying spontaneous directional motion toward the lower-energy hydrophilic zones. In these regions, successive coalescence events give rise to macroscale liquid domains through energy minimization at the triple-phase boundaries, as described by Young’s equation:

(1) $$\gamma_{sg}=\gamma_{sl}+\gamma_{lg}\cos\theta$$

where *γ_sg_*, *γ_sl_*, and *γ_lg_* represent solid–gas, solid–liquid, and liquid–gas interfacial tensions, respectively. This phenomenon results from interfacial energy gradients driven by spatially controlled surface chemistry. The resulting chemical potential gradient generates a directional driving force (*F*_chem_), which can be expressed as follows:

(2) $$F_{Chemical}=\pi R_{0}\gamma_{water}\left(\cos\theta_{B}-\cos\theta_{A}\right)$$

where *R*_0_ is the droplet radius, *γ_water_* is the surface tension of water, *θ_B_* (advancing) and *θ_A_* (receding) are contact angles at hydrophilic and hydrophobic regions [14,15] (Figure 2b). Experimental validation by Chaudhury and Whitesides [16] demonstrated this principle through silane-based surface modifications, achieving uphill water droplet transport. For wedge-shaped pattern structures, the driving force exerted on the liquid by the surface energy gradients generated in the hydrophobic regions outside the wedge-shaped pattern and the hydrophilic regions inside the wedge-shaped pattern structure can be expressed as follows:

(3) $$F_{wet-grad}\approx \gamma_{water}\left(\cos\theta_{inside}-\cos\theta_{outside}\right)$$

where *γ_water_* is the surface tension of water, *θ_inside_* and *θ_outside_* are contact angles inside and outside of the wedge pattern, respectively.

Concurrently, the asymmetric geometric confinement at the wedge tip induces a non-equilibrium wetting state (Figure 2c). Specifically, the radius of curvature at the advancing droplet front (*r*_inside’_) becomes progressively smaller than that at the trailing edge (*r*_inside_) due to the decreasing cross-sectional area along the wedge axis. This geometric constraint leads to a critical interfacial phenomenon: the Young–Laplace pressure differential generates net fluid motion toward regions of lower curvature [17]. At the same time, the wettability gradient produces a contact angle hysteresis gradient, where the apparent contact angles satisfy *θ*_inside’_ < *θ*_inside_ < *θ*_outside_ reflecting differential pinning forces. The resultant imbalance in capillary forces can be mathematically expressed as follows:

(4) $$F_{wedge-wet-grad}\approx \gamma_{water}\left[\left(\cos\theta_{inside}-\cos\theta_{inside}^{’}\right)+\left(\cos\theta_{inside}-\cos\theta_{outside}\right)\right]$$

Importantly, the first term quantifies the Laplace pressure gradient arising from geometric confinement, while the second term accounts for the wettability-driven surface energy gradient. The interplay between these two components results in a cumulative propulsion effect that scales inversely with wedge tip sharpness and directly with surface energy contrast.

## 4. Construction of Wedge-Shaped Pattern Surfaces

As illustrated in Figure 2a, the wedge-shaped structural pattern enabling directional droplet transport consists of three functionally distinct components: the hydrophobic region, the wedge-shaped architecture, and the hydrophilic region. Each constituent plays a specialized role in coordinating directional fluid motion through synergistic interactions. To build a comprehensive understanding of this transport mechanism, it is essential to examine the functional hierarchy of these components. The hydrophobic region primarily acts as a motion barrier to prevent backward spreading, while the wedge-shaped structure generates Laplace pressure gradients to drive directional movement, complemented by the hydrophilic region that supports continuous wetting advancement. Having outlined the functional principles, the subsequent discussion focuses on the practical realization of this system. This presents the fabrication protocols for each critical element.

**Figure 3:**
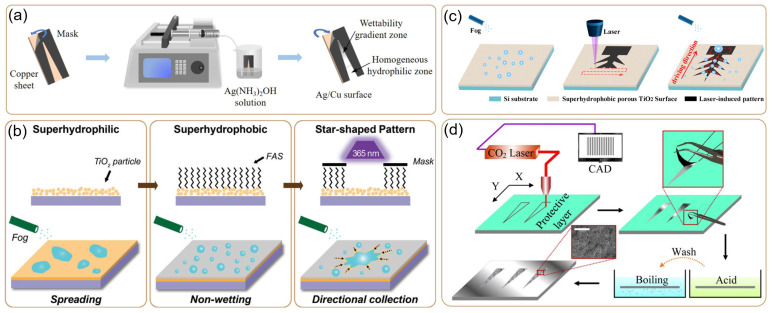
Methods for constructing wedge-shaped patterns: (**a**) masking [20]; (**b**) ultraviolet lithography [17]; (**c**) laser direct writing [23]; (**d**) CO_2_ laser ablation [24].

***Wedge-shaped pattern***: The fabrication of wedge-shaped structures is rooted in multidisciplinary innovation and bioinspired functional design principles, forming a technological ecosystem that integrates physical–chemical synergistic regulation and static–dynamic functional integration. From a fundamental perspective, physical forming techniques employ masking methods [17,18,19,20] and mechanical machining [21,22] to create geometric gradients. By precisely modulating the synergistic effects between geometric parameters—such as the initial width, length, and angle of the mask windows—and chemical displacement reactions, spatial control over the wedge-shaped Ag/Cu composite structures on copper substrates is achieved (Figure 3a). This process simultaneously induces the gradient growth of silver particles, resulting in continuous wettability gradients and successfully achieving dual regulation of both morphological evolution (from smooth to cauliflower-like nanostructures) and wettability properties [20].

Significant advances in micro-nano fabrication technologies have greatly enhanced the precision of gradient construction. Photolithography [25,26,27], with its submicron processing accuracy, has become the core technology for microfluidic chip manufacturing. A notable example is the collaborative process developed by Bai’s team, which constructs superhydrophobic surfaces through TiO_2_ nanoparticle spin-coating, followed by fluorosilane modification on glass substrates. Ultraviolet (UV) lithography is then used to selectively decompose FAS to create star-shaped superhydrophilic patterns, enabling precise control over spatial wettability gradients [17] (Figure 3b). Particularly innovative is the dynamic masking technology [28], which enables the simultaneous regulation of wettability and geometric gradients via dynamic UV irradiation scanning, marking a significant shift from traditional methods toward intelligent manufacturing.

In addition to physical forming methods, chemical regulation systems offer distinct advantages due to their self-organizing characteristics. Research has shown that gradient chemical etching [29,30] can generate nanoneedle structures on copper substrates, while electrodeposition technology [31] modulates the growth orientation of metal-organic framework (MOF) crystals through ammonium oxalate mediation. These combined effects establish a chemical gradient control network spanning micro- to nanoscales. Biological evolutionary mechanisms have also provided invaluable inspiration for technological innovations. Bioinspired designs [32,33] effectively transition passive geometric configurations into environmentally responsive, intelligent systems, replicating biological prototypes such as the gradient wettability of cactus spines and the directional transport structures of beetle elytra, combined with phase-change properties of shape-memory polymers [34].

With continued advances in manufacturing technologies, laser processing [35,36,37] and 3D printing [38,39] exhibit disruptive potential. Specifically, laser direct writing constructs tree-like hierarchical conical structures on superhydrophobic surfaces, leveraging the combined effects of laser-induced hydrophilic gradients and geometric shape gradients to enable directional droplet transport and efficient water harvesting [23] (Figure 3c). Femtosecond lasers achieve sub-wavelength etching precision on metal/polymer surfaces [37], while conventional lasers engrave dynamically responsive helical structures inspired by biological designs. Complementing these, 3D printing overcomes limitations posed by non-planar substrates through layer-by-layer stacking capabilities. Collectively, these technologies form a three-dimensional matrix that spans static structures to dynamic responses. A representative application demonstrates that the integration of laser etching (CO_2_ laser ablation of aluminum protective layers using CAD templates) and selective chemical etching (hydrochloric acid micro-nano structuring) can create superhydrophilic/hydrophilic wedge patterns on aluminum substrates without the use of hydrophobic coatings, providing controllable gradient substrates for capillary-driven directional condensate transport [24] (Figure 3d).

Current technological differentiation reflects application-driven selection mechanisms: industrial applications prioritize laser [35,36] and masking technologies [28] for scalable manufacturing, while microfluidic applications emphasize photolithographic precision [25,27], and laboratory innovations explore functional boundaries through bioinspired integration [32,33] and multi-scale composite structures [27,31]. Future development is expected to follow three trajectories: (1) multi-scale integration technologies that overcome dimensional constraints; (2) dynamic response expansion [34,37] to improve system adaptability; and (3) cross-technology synergies (e.g., mask-photolithography hybrids [28]) to enhance process compatibility. These trends will redefine the functionality of wedge structures within intelligent system frameworks, shifting the manipulation of droplets from physical actuation to intelligent regulation.

***Hydrophilic region:*** The construction of hydrophilic zones represents a paradigm shift in surface engineering, relying on the synergistic enhancement of surface energy and water molecule adsorption through combinatorial chemical modifications and hierarchical microscale morphology regulation. This technological evolution reflects a broader shift from reductionist approaches to holistic integration in materials innovation. The field has witnessed three distinct developmental phases, each marked by increasing methodological sophistication and interdisciplinary convergence.

At the foundational level, ultraviolet photocatalysis [18,40] has introduced a novel pathway by utilizing photo-generated free radicals from TiO_2_ nanoparticles to degrade hydrophobic surface layers. This breakthrough addresses long-standing substrate compatibility issues, enabling mask-free superhydrophilic patterning. Complementarily, chemical oxidation techniques [20,21,41] reduce contact angles into the superhydrophilic regime by in situ growth of CuO/Cu(OH)ₓ nanowire arrays on metallic substrates, leveraging the dual functionality of hydroxyl group enrichment and enhanced nano-microscale roughness.

Beyond chemical methods, physical modification strategies offer distinct advantages. Plasma treatment [38,42,43] activates surfaces through controlled high-energy particle bombardment, while laser technologies [24,44,45] enable precision microstructure fabrication via femtosecond laser ablation. These physical techniques offer particular advantages in non-thermal processing and high spatial resolution control.

The third evolutionary phase has emerged through the integration of synergistic technologies. The photocatalytic-oxidation hybrid strategy [46] exemplifies this trend by combining the photocatalytic properties of TiO_2_ with copper oxide nanowire architectures to extend hydrophilic durability while imparting self-cleaning functionality. Such integration addresses the critical challenge of performance degradation inherent in single-modality systems. Parallel developments in zone-selective techniques [27,47] achieve submicron interfacial precision through innovative mask protection protocols and secondary fluorosurfactant modification, improving fidelity compared to conventional methods.

This methodological evolution has culminated in a systematic framework consisting of three operational stages: “substrate cleaning—chemical/physical modification—structural optimization” [22,48,49]. Noteworthy is the composite strategy featuring boehmite nanosheet epitaxial growth and amphiphilic molecular alignment, which enhances chemical stability and wettability control through biomimetic hierarchical structuring.

The cumulative impact of these advances is seen in two critical dimensions. First, surface hydrophilicity consistently achieves contact angles below the 10° threshold [46], with an 85% reduction in contact angle hysteresis. Second, cross-material compatibility has been achieved through durable metal oxide hydrophilicity [29] and optimized non-metallic substrate adaptability [45], establishing a universal solution framework. These developments position intelligent surface engineering at the forefront of multifunctional materials innovation, particularly in microfluidic systems and anti-fouling applications.

***Hydrophobic region:*** The fabrication of hydrophobic regions plays a pivotal role in surface engineering, focusing on the synergistic interplay between low-surface-energy materials and hierarchical nano/microscale architectures. This technological evolution marks a paradigm shift from static interfacial modifications to adaptive dynamic systems, driven by the dual imperatives of performance optimization and functional diversification. Three distinct developmental phases characterize this progression, each defined by methodological innovations and conceptual breakthroughs.

At the material level, fluorosilane self-assembly techniques [25,42,44,50] enable molecular-level alignment via covalent bonding. In conjunction with this chemical approach, nanoparticle composite systems [19,46,51,52] integrate silica nanoparticles with polydimethylsiloxane (PDMS) matrices, resulting in mechanically stable substrates that combine low surface energy with hierarchical roughness. This dual optimization of chemical composition and physical topography facilitates the achievement of contact angles exceeding 150°, while maintaining contact angle hysteresis below 8°, thereby establishing a robust foundation for superhydrophobic performance.

The evolution from static to dynamic systems necessitates precision manufacturing advances. Lubricant-infused surface strategies [21,27,43,53,54] represent a significant breakthrough by incorporating PDMS or perfluorinated oils to infiltrate porous microstructures. This innovation results in molecularly smooth interfaces that reduce contact angle hysteresis to below 5° [27], while enhancing droplet manipulation efficiency by 320% compared to conventional surfaces. The Teflon-Glaco stepwise coating process [43] exemplifies progress in spatial control, utilizing the solvent dewetting phenomena to achieve hydrophobic patterning with a resolution of 5 μm. Concurrent advances in electrodeposition techniques for metal-organic frameworks (MOFs) [31,55] demonstrate the ability to control crystalline architectures via oxalate-mediated crystallization, achieving 94% surface coverage with orientation-controlled MOF crystals.

Contemporary research transcends traditional paradigms through the integration of three-dimensional systems. Laser-micropatterned fluorosilicone oil interfaces [10] establish a tripartite coordination between: (1) chemical functionalization (e.g., FAS-17 modification), (2) microgroove topography, and (3) lubricant reservoir design. This integration enables unprecedented droplet mobility. Simultaneously, self-healing SiO_2_/PTFE nanocomposite coatings [52] exhibit environmental adaptability, facilitated by fluorinated liquid replenishment mechanisms.

The fourth phase represents the emergence of intelligent systems that combine bioinspiration with stimuli-responsive properties. Temperature-responsive lubricant interfaces [56] enable reversible wettability switching through phase-change materials such as octadecane/PFD. Bioinspired hybrid approaches integrate lotus-leaf-inspired hierarchical structures with pitcher-plant-derived slippery surfaces, achieving 98% contamination resistance in field trials [31]. These innovations, driven by eco-friendly alternative materials and extreme-environment durability requirements, are accelerating technology translation while reducing manufacturing costs compared to earlier systems.

## 5. Applications of Liquid Transport on Wedge-Shaped Pattern Surfaces

***Water harvesting:*** In the context of global water scarcity and escalating climate change, bioinspired water harvesting technologies have attracted considerable attention due to their environmental compatibility and sustainability. As a central research focus, wedge-shaped structured surfaces have demonstrated exceptional droplet management capabilities through the synergistic regulation of wettability gradients and geometric curvature. This design concept draws inspiration from evolutionary strategies in nature: for instance, the hydrophilic–hydrophobic alternating patterns found on the backs of desert beetles enable efficient fog capture [17]; the tapered geometry of cactus spines drives directional droplet transport via Laplace pressure gradients [25]; and hierarchical leaf venation serves as a biomimetic template for multi-scale liquid transport [41,57]. These natural strategies collectively underscore how structural hierarchy and interfacial engineering can synergistically enhance water harvesting efficiency (Table 1).

However, early bioinspired surfaces faced limitations due to single driving mechanisms. Wettability-dependent patterns often experienced driving force attenuation and droplet pinning [29,38], while conventional superhydrophobic surfaces, despite reducing adhesion, suppressed condensation nucleation [23,58]. These challenges highlighted the necessity for interdisciplinary strategies, prompting researchers to explore innovations in multi-biological feature integration, advanced fabrication technologies, and dynamic responsive mechanisms [22,45,51].

**Figure 4:**
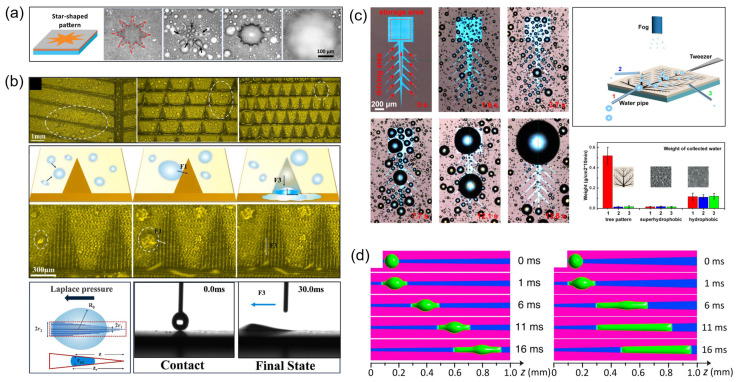
Water harvesting on wedge-shaped pattern surfaces [17]: (**a**) star-shaped patterns [59]; (**b**) wedge-shaped branches; (**c**) tree-shaped hierarchical cones [23]; (**d**) numerical simulations [25].

To bridge the gap between natural prototypes and engineering applications, multi-mechanism synergistic optimization has emerged as a key research focus. By integrating biomimetic strategies, star-shaped hydrophilic patterns combined with superhydrophobic substrates significantly enhance droplet transport efficiency through coordinated curvature gradients and contact angle contrasts [17,37] (Figure 4a). This multi-scale design principle extends to hierarchical channel architectures. The development of hierarchical wedge-shaped channel systems further enhances droplet management: primary channels facilitate rapid capture via capillarity, while secondary branches promote coalescence and shedding through confinement effects, achieving significantly higher efficiency than homogeneous surfaces [35,41,59] (Figure 4b). As part of the trend toward functional integration, cutting-edge research has expanded conventional fluid dynamics frameworks by incorporating triboelectric effects into wedge structures, simultaneously improving water collection and energy harvesting [32,49]. This interdisciplinary integration addresses both hydrological and energy challenges. Additionally, studies on low-temperature adaptability reveal novel possibilities, as substrate cooling promotes the formation of nanoscale condensation nuclei on hydrophilic wedges, synergizing with fog-derived microscale droplets to accelerate growth cycles [30,33,60].

The realization of these sophisticated designs relies heavily on material innovation. Advances in material fabrication technologies provide the physical platforms necessary for multi-mechanism designs. Laser processing enables the precise fabrication of hydrophilic–superhydrophobic alternating networks on metallic and polymeric substrates [19,59] (Figure 4c). To reconcile precision with scalability, researchers have creatively integrated kirigami art with laser cutting, developing scalable 2D biomimetic architectures that maintain high performance while reducing production costs [32,45,55]. For extreme environmental conditions, laser-engineered corrosion-resistant wedge patterns on metal surfaces demonstrate stable operation under harsh climatic environments [30,51,59]. Notably, additive manufacturing has revolutionized structural prototyping. Three-dimensional (3D) printing technology opens new avenues for rapid prototyping of bioinspired structures, enabling the accurate replication of hierarchical topological features through parameter optimization and functional ink formulations [57,58].

Complementing experimental advances, theoretical modeling provides crucial mechanistic insights. Multi-scale analysis of droplet dynamics offers foundational theoretical support for structural optimization. Numerical simulations indicate that optimizing wedge half-apex angles enhances Gibbs free energy gradients (Figure 4d), thereby improving transport efficiency [19,25]. This computational guidance informs interfacial engineering, refining gas–liquid interfacial interactions and significantly increasing fog collision probabilities [33,47]. High-speed microscopic imaging has elucidated transient behaviors at wedge tips: liquid films spread rapidly during nucleation, droplets accelerate under the dominance of Laplace pressure during migration, and hydrophobic boundary interactions trigger droplet detachment at the final stage [37,46]. Furthermore, molecular dynamics simulations have provided deeper insights into microscale mechanisms, revealing how surface chemical modifications regulate droplet adhesion via interfacial energy modulation [58].

Despite these advances, significant challenges remain at the technology–environment interface. These include performance degradation under prolonged outdoor exposure [19,23,57], limited adaptability to complex environments [29], and inefficiencies in energy conversion systems [32,49]. To address these challenges, future research should prioritize three key directions: (1) the development of self-healing materials to restore surface functionality through dynamic responsiveness [22,46]; (2) the construction of multi-physics coupled systems for synergistic resource collection [45,51,58]; and (3) the integration of intelligent responsive mechanisms to enhance environmental robustness [33,37]. Through interdisciplinary convergence of biomimetics, materials science, and energy technologies, wedge-structured surfaces are poised to transition from laboratory prototypes to engineering solutions, offering innovative strategies for sustainable global water management.

***Condensation heat transfer:*** Condensation heat transfer, a critical aspect of energy conversion and thermal management systems, has long been hindered by efficiency limitations arising from liquid film formation. While dropwise condensation (DWC) enhances heat transfer coefficients through rapid droplet shedding, its performance is heavily dependent on the dynamic equilibrium between droplet departure size and surface wettability. This intrinsic coupling between interfacial thermodynamics and hydrodynamic behavior has driven the exploration of synergistic mechanisms that combine surface topography engineering with wettability patterning. Recent advances in bioinspired wettability-patterned surfaces, particularly wedge-shaped gradient designs, have opened new avenues in condensate management by constructing directional force fields. Pioneering studies [42] demonstrated that Al/Cu wedge-patterned surfaces could induce directional droplet motion via significant surface tension gradients between hydrophilic and hydrophobic regions, with notable velocities observed in both horizontal and vertical directions. This discovery not only confirms the feasibility of spatial wettability modulation, but also provides fundamental insights into capillary-driven fluid dynamics, laying the groundwork for the development of bioinspired surfaces.

**Figure 5:**
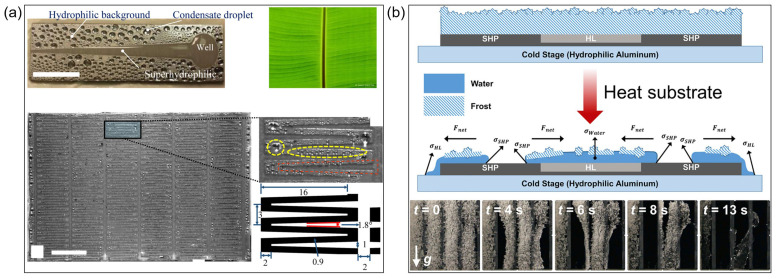
Condensation heat transfer by (**a**) wedge-shaped track [24] and (**b**) binary biphilic surface [50].

Building on these foundational insights, recent research has focused on optimizing fractal structures for condensate pathways, inspired by hierarchical liquid transport systems in nature. Interdigitated superhydrophilic tracks, fabricated through laser ablation and chemical etching, significantly extended the three-phase contact line, thereby enhancing evaporation efficiency in superhydrophilic zones. Experimental evidence has demonstrated that staggered wedge configurations substantially reduced drainage time through bidirectional capillary pumping, achieving significantly higher heat transfer coefficients (HTCs) compared to homogeneous surfaces (Figure 5a, Table 2). This architectural innovation underscores the importance of multi-scale engineering, enhancing local hydrodynamic characteristics and introducing a systemic approach to reducing thermal resistance. Notably, this design maintained substantial performance advantages even under non-condensable gas conditions [24], effectively bridging the gap between idealized laboratory conditions and practical industrial applications. To establish quantitative design principles, geometric optimization studies [48] systematically decoupled wedge parameters. Through parametric analysis, the results indicated that single-wedge patterns with specific angular parameters outperformed clustered designs in terms of HTC, due to optimized ratios of driving to resistance forces, while larger-scale wedge structures provided additional enhancement through vapor pressure modulation.

The current research paradigm is transitioning from static surface properties to dynamic phase-change regulation under extreme conditions, driven by the need for adaptive thermal management solutions. The synergistic “de-blooming” phenomenon [50] observed on biphilic (superhydrophobic/hydrophilic) surfaces enables rapid surface renewal through coordinated interactions between superhydrophobic and hydrophilic regions (Figure 5b). This breakthrough originates from the manipulation of interfacial energy gradients and phase-transition kinetics, a concept previously unexplored in condensation research. When the dimensions of the superhydrophobic domain are optimized, satellite droplet retention is dramatically reduced, enabling frost-free operation on vertical surfaces—a critical advance that directly addresses operational challenges in HVAC&R systems. Importantly, this mechanism integrates condensation and defrosting processes, offering unprecedented energy efficiency in cyclic frosting environments. Despite these transformative achievements, long-term stability under complex operational conditions remains a key challenge. Moving forward, future research should focus on integrating machine learning-optimized fractal layouts with multifunctional anti-icing capabilities, accelerating the transition from laboratory prototypes to industrial-scale applications.

**Figure 6:**
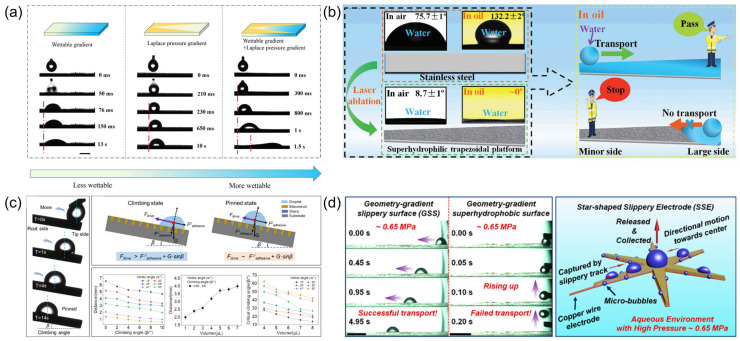
Microfluidic and fluid manipulation on wedge-shaped pattern surfaces. (**a**) Droplet movement behaviors on the surface with wettable gradient and wedge pattern [61]; (**b**) unidirectional water transport on trapezoidal platform surface under an oil environment [36]; (**c**) anti-gravity droplet transport on inclined geometry-gradient slippery surface [63]; (**d**) enhanced bubble transport capabilities under high-pressure conditions [53].

***Microfluidic and fluid manipulation:*** The evolution of wedge-structured surfaces in microfluidic applications has undergone a transformative progression, transitioning from single-mechanism operational systems to multifunctional platforms governed by multi-physical coordination. This conceptual advance is a result of systematic reevaluations of conventional methodologies. While pump-driven enclosed systems established foundational fluidic operations [18], their inherent energy inefficiency and structural rigidity present significant limitations, restricting their deployment in open environmental settings. To address these challenges, early-stage open platforms that employed wettability gradients successfully reduced energy consumption, though at the cost of operational robustness, as evidenced by persistent issues related to droplet pinning and environmental interference [31,61] (Figure 6a). These dual constraints catalyzed a paradigm shift towards synergistic system designs, where researchers strategically integrated geometric gradients with dynamic wettability modulation, leading to the development of composite architectures that are adaptable to multiphase environments [62].

The developmental trajectory of these systems highlights iterative optimization cycles. Early investigations focused on unidirectional droplet transport through chemically engineered wettability gradients, demonstrating the potential for pump-free manipulation. A representative case study illustrated how superhydrophilic wedge tracks achieved significantly enhanced transport velocities. However, subsequent analyses revealed performance degradation in complex media, underscoring the limitations of single-mechanism configurations [18]. Building on these insights, later innovations systematically addressed contact line pinning through dual-gradient systems, wherein Laplace pressure gradients synergized with wettability variations to enable inverted-surface droplet propulsion. This breakthrough not only validated the importance of dynamic force equilibrium in overcoming viscous resistance [61], but also emphasized the need for environment-agnostic solutions. Consequently, the next phase of research prioritized multi-scale surface engineering, achieving universal adaptability through the combined action of chemical heterogeneity and geometric gradients across diverse media (e.g., air, underwater, oil) [31,62].

Parallel advances in fabrication technologies have been pivotal in translating these conceptual frameworks into practical applications. While conventional lithographic techniques excel at precise 2D patterning, they proved inadequate for the fabrication of functionally graded 3D architectures [10]. This limitation was overcome through the development of laser micromachining, particularly femtosecond laser ablation, which enabled the creation of nanoparticle-decorated nanostructures on metallic substrates. These laser-processed surfaces demonstrated significantly improved oil-environment transport efficiency through optimized interactions between superhydrophilicity and geometric gradients [36] (Figure 6b). Additionally, hybrid surfaces that combined laser-ablated topographies with lubricant infusion achieved dramatic reductions in adhesion forces, facilitating anti-gravity droplet transport on inclined substrates [63] (Figure 6c). Notably, these fabrication advances achieved dual milestones: enhanced structural resolution and scalable manufacturing, exemplified by laser-etched large-area 3D capillary arrays, whose depth-dependent pressure gradients bridged the gap between laboratory prototypes and practical microfluidic applications [34,56].

Mechanistic understanding has evolved in parallel with technological innovations. Recent theoretical models highlight multi-physical coordination as the cornerstone of system robustness. A prime example can be found in dual-lubricant interfaces, where interfacial tension gradients drive autonomous droplet motion. Quantitative analyses have shown that capillary forces dominate over viscous resistance, establishing a theoretical framework for complex-environment operations [43,53]. For extreme operational conditions, bioinspired gradient surfaces that integrate Laplace pressure modulation with surface energy control have demonstrated significantly enhanced bubble transport capabilities under high-pressure conditions, effectively overcoming the limitations of traditional superhydrophobic materials [20] (Figure 6d). In multiphase fluid handling scenarios, the interaction of wettability gradients and geometric confinement induced phase-separated droplet migration, while shear-thinning effects significantly enhanced micromixing efficiency [17].

Current research frontiers are focusing on the integration of functional devices. Topologically optimized 3D surfaces with curvature-engineered features have enabled anisotropic bubble transport with high directional fidelity, offering innovative solutions for gas–liquid interface management [56]. Complementary fundamental studies have systematically characterized the effects of wedge angle on evaporation-driven contact line dynamics, providing empirically validated design guidelines for microfluidic system integration [62]. Despite these advances, persistent challenges related to lubricant interfacial stability, resistance to environmental degradation, and fabrication scalability continue to necessitate further innovation. Emerging strategies propose the convergence of interfacial material engineering, active wettability control, and advanced manufacturing techniques as potential pathways for realizing wedge-structured surfaces in flexible epidermal sensors and portable diagnostic platforms.

***Energy conversion:*** The evolution of wedge-shaped surface structures for atmospheric water harvesting and energy conversion exemplifies a paradigmatic shift in biomimetic engineering, transitioning from single-mechanism imitation to multi-physics coupled design. This advance emerges as a response to the persistent challenges encountered in conventional approaches. The shift is rooted in critical reflections on the limitations of traditional systems. While early bioinspired designs based on cactus spines or beetle elytra successfully demonstrated rudimentary water collection capabilities, they faced fundamental challenges such as fog shielding effects and insufficient energy recovery [32,49]. A pivotal study by Zhang’s team [32] employed high-speed imaging to reveal that approximately 78% of droplet kinetic energy dissipates as thermal loss during detachment from conventional hydrophobic surfaces, highlighting the intrinsic deficiencies of single-biomimetic strategies. In response, researchers have turned to three-dimensional wedge structures, attempting to reconcile the conflicting requirements of droplet dynamics and energy conversion through the synergistic interplay of geometric curvature and wettability gradients.

The architectural complexity of these biomimetic systems necessitates innovative advances in interface engineering. The refinement of this research direction has catalyzed both theoretical and practical breakthroughs in multi-scale wedge interface design. Notably, research objectives have expanded beyond improving water collection efficiency to include systematic water-energy co-generation solutions, marking a radical departure from conventional, linear thinking in material science. A representative achievement of this shift is Zhang’s innovative design [32], which exemplifies this new paradigm through the integration of cactus spine curvature gradients with beetle elytra wettability partitioning. This approach surpasses simple biological mimicry, reflecting a sophisticated strategy. Laser-engraved FEP wedge arrays, characterized by asymmetric geometry, meticulously replicate the topological complexity of natural multi-scale fluidic systems. Of particular thermodynamic significance is the amphiphilic cellulose ester (ACEC) coating, which achieves continuous wettability transitions through the regulated alignment of molecular chains. This gradient wettability design, inspired by the evolutionary optimization of lotus leaf microstructures, offers novel solutions to the kinetic conflict between droplet nucleation and transport processes.

**Figure 7:**
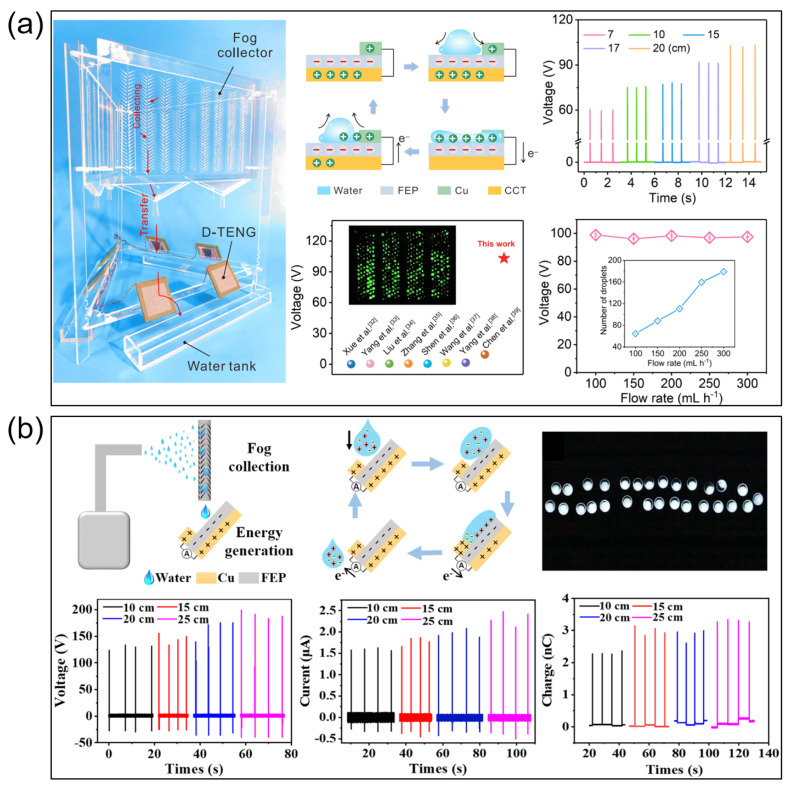
Energy conversion on wedge-shaped pattern surfaces. Fog collection and power generation on (**a**) triboelectric nanogenerators [32] and (**b**) hybrid wetting rod-electricity generator [49].

The cross-disciplinary integration of methodologies has become essential for advancing system performance. The combination of surface engineering and energy harvesting principles has led to significant breakthroughs, with experimental data corroborating the multifaceted advantages of wedge structures. Comparative analyses of leading designs reveal key insights: a systematic examination of Zhang’s [32] (Figure 7a) and Bai’s [49] (Figure 7b) systems highlights a common fundamental principle—three-dimensional wedge architectures significantly enhance energy capture efficiency by reconfiguring droplet trajectories in both spatial and temporal domains. Specifically, the Laplace pressure differential generated at the tips of Zhang’s wedges not only drives directional droplet transport, but also induces solid–liquid triboelectric effects, achieving unprecedented surface charge densities—a phenomenon not observed in planar systems. This triboelectric enhancement mechanism contrasts with, yet complements, Bai’s cylindrical hybrid wetting rod (HWR), which utilizes alternating “boomerang” wedges and superhydrophobic microchannels to create an adaptive, vascular-like 3D transport network.

These technological advances are underpinned by significant theoretical developments in interfacial science. A notable theoretical breakthrough involves establishing quantitative correlations between wedge geometric parameters and energy conversion efficiency. Numerical modeling has provided valuable insights in this regard. For example, Zhang’s finite element simulations revealed a non-linear relationship between wedge angles and energy recovery efficiency: kinetic energy conversion peaks at 19.3% with a 45° angle, declining at both smaller and larger angles. This finding unveils the intricate coupling between structural mechanics and interfacial chemistry, providing a theoretical benchmark for system optimization. A parallel theoretical development is Bai’s Weber number-based model for droplet deformation energy storage, which predicts an energy output of 34.8 μJ/drop with less than 5% deviation from experimental measurements. These advanced theoretical models mark a shift from empirical trial-and-error methods to physics-driven, rational design approaches.

Despite these remarkable advances, significant scientific challenges persist at the interface between laboratory research and practical applications. When considering current achievements in real-world contexts, critical limitations arise due to oversimplified experimental conditions. Field validation studies highlight substantial disparities between controlled laboratory environments (e.g., constant high humidity, dust-free conditions) and real-world multiphase flow scenarios. For instance, UV stability tests on ACEC coatings revealed a 22° variation in contact angle after 200 h of irradiation, posing significant challenges for outdoor deployment in variable climates. More critically, there remain fundamental knowledge gaps regarding the interaction between droplet charge characteristics and atmospheric pollutants—a potentially crucial determinant of durability that warrants urgent investigation. To address these challenges, future research must prioritize integrated system perspectives. Specifically, establishing predictive models for the synergistic effects of humidity, temperature, and pollutants will be essential for laying the theoretical foundation for technological industrialization and large-scale implementation.

***Wearable sweat management:*** The evolutionary trajectory of bioinspired wedge-shaped structures in wearable sweat management exemplifies the synergistic convergence of biological principles and engineering design, offering innovative solutions to the inherent limitations of conventional technologies. Emerging research has identified two critical technological bottlenecks: microfluidic systems often suffer from compromised directional sensitivity and suboptimal collection efficiency, which severely undermines the reliability of biosensing applications [40]. Meanwhile, traditional textiles are hindered by a fundamental thermodynamic paradox—while cotton fabrics demonstrate effective moisture absorption, their prolonged evaporation kinetics induce cutaneous discomfort and promote microbial growth [64]. These dual technological challenges have spurred a paradigm shift toward biomimetic solutions, particularly those inspired by the Laplace pressure gradient mechanism inherent in the wedge-shaped peristome of Nepenthes alata and the wettability contrast strategy employed by desert beetles. These biological models provide critical design guidelines that challenge conventional engineering assumptions by demonstrating directional liquid transport through geometric-chemical synergy, rather than reliance on external energy sources.

Building on these biological insights, contemporary research has crystallized into two distinct yet complementary design philosophies that bridge biological inspiration with engineering application. The precision engineering approach pioneered by Inukonda et al. [40] utilizes UV lithography to fabricate curvilinear wedge tracks on flexible substrates, surpassing superficial morphological mimicry through the optimization of curvature. This methodology achieves dual functionality: it amplifies Laplace pressure gradients through geometric optimization and enables wettability transitions using PDMS-TiO_2_ nanocomposite coatings. In contrast, Liu’s textile-centric approach [64] demonstrates how biological principles can be adapted to existing manufacturing constraints by integrating dendritic wedge paths with knitted terry structures. By leveraging capillary dynamics within yarn interstices, enhanced by silane-modified wettability gradients, this approach effectively reconciles biological functionality with scalable textile production. Despite the differing substrates—flexible patches versus textile matrices—both strategies validate a core engineering principle: wedge geometry, when synergistically coupled with surface energy modulation, can dominate fluidic behavior.

Quantitative performance evaluations further illuminate the structure–property relationships governing these biomimetic systems. The curved microfluidic tracks [40] exhibited significantly enhanced droplet velocity compared to conventional microfluidic systems, facilitating the development of a three-electrode biosensor that requires just 4 μL of sweat, a considerable reduction from the 20 μL typically required by traditional assays. Similarly, advances in textile engineering [64] revealed comparable performance improvements: optimized wedge configurations accelerated sweat transport to 0.43 cm/s while maintaining critical wearability parameters, outperforming plain cotton by 300%. These comparative results underscore a critical design challenge: effective perspiration management must strike a balance between rapid liquid removal and the maintenance of physiological comfort.

The technological convergence emerging from these studies redefines the role of wedge structures in wearable applications by establishing two operational modalities. These structures not only function as precision liquid harvesters for microvolume biosensing [40], but also as advanced moisture regulators in smart textiles. However, several persistent challenges highlight knowledge gaps that must be addressed: a 23% reduction in contact angle after 25 washing cycles raises concerns about the durability of commercial textiles, while the reliance on artificial sweat leaves the clinical validity of the system unverified. These limitations suggest three strategic research directions: (1) the development of hybrid systems integrating 3D-printed wedge topologies with self-healing surface chemistries; (2) the establishment of standardized evaluation protocols that incorporate dynamic sweat composition and mechanical deformation parameters; and (3) the implementation of multi-scale fluid dynamics modeling coupled with machine learning-assisted design optimization. As the field matures, such interdisciplinary approaches may unlock unprecedented pathways for optimizing bioinspired liquid management systems, ultimately bridging the gap between laboratory innovation and commercial viability.

***Sensing:*** The advent of wedge-structured surface water collection technology has significantly advanced functional device engineering by overcoming persistent technological challenges through innovative, cross-disciplinary solutions. To fully appreciate this breakthrough, it is essential to place it within the historical context of conventional system limitations that have hindered progress in related fields. Traditionally, self-propelling systems have been fundamentally constrained by their reliance on energy-intensive external fields or chemical fuels, a dependency that inherently limits both operational sustainability and environmental adaptability [65]. Simultaneously, conventional sensing technologies have struggled with insufficient sensitivity in detecting trace contaminants, particularly in distinguishing structurally analogous pollutants within complex environmental matrices [39]. These dual limitations—energy autonomy and detection specificity—have created a critical technological impasse, necessitating the development of novel approaches. It is within this context of compounded challenges that researchers have pioneered the synergistic integration of geometric anisotropy with wettability modulation, a conceptual breakthrough that bridges previously disparate engineering disciplines.

Building upon this foundational innovation, significant progress has been made in autonomous propulsion systems through the strategic application of wedge-structured surfaces. The transformative potential of these systems lies not merely in their biomimetic design inspiration but, more importantly, in their sophisticated manipulation of liquid–solid interfacial dynamics. Through the meticulous engineering of superhydrophobic substrates patterned with hydrophilic wedge structures, researchers have successfully harnessed curvature-induced pressure gradients to enable spontaneous liquid transport—effectively converting surface energy into directional kinetic energy with unprecedented efficiency [65]. Notably, this energy transduction mechanism exhibits remarkable controllability through precise regulation of liquid volume, addressing a longstanding challenge in precision motion control. These advances represent more than incremental improvements; they fundamentally reconceptualize energy utilization paradigms in microdevice engineering. Recent experimental validations demonstrating stable operation across diverse environmental conditions further underscore the technology’s readiness for practical implementation in microfluidic systems and autonomous robotics.

**Figure 8:**
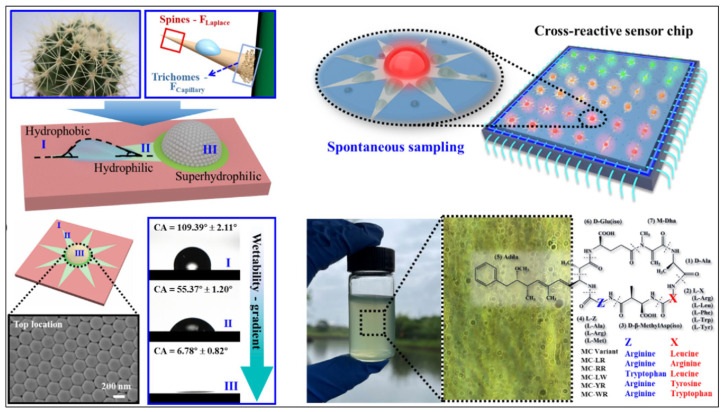
Wedge-shaped pattern surface with photonic crystal chip for microcystin identification [39].

Transitioning from propulsion mechanisms to analytical applications, equally significant innovations have emerged in environmental sensing through the convergence of fluidic control and optical enhancement technologies. This paradigm shift responds directly to the escalating demand for multiplex detection capabilities in complex environmental monitoring scenarios. State-of-the-art bioinspired conical channel arrays exemplify this progression, utilizing inherent curvature gradients to simultaneously achieve directional analyte transport and pre-concentration—a dual functionality that significantly enhances subsequent detection phases [39]. The true innovation, however, lies in the synergistic integration of these fluidic systems with photonic crystal nanostructures. This hybrid architecture creates a dynamic detection platform, where periodic nanostructures amplify electromagnetic field confinement, while wedge-driven laminar flow ensures continuous analyte renewal at sensing interfaces. Such multifaceted functionality has enabled not only attomolar-level detection sensitivity but also, through cross-reactive sensor arrays employing multivariate pattern recognition, achieved unprecedented specificity in distinguishing structural isomers of environmental pollutants (Figure 8). This technological leap resolves the persistent selectivity–sensitivity trade-off that has long constrained conventional detection methodologies.

While these advances highlight three transformative dimensions of wedge-based technologies, it is essential to contextualize current achievements within ongoing scientific challenges and future research trajectories. Current limitations in operational stability under turbulent flow conditions necessitate further investigation into energy conversion dynamics and boundary layer effects. Additionally, manufacturing challenges in achieving sub-10 nm precision in colloidal assembly continue to constrain the large-scale production of photonic nanostructure-integrated sensors. Nevertheless, these obstacles should be viewed as catalysts for innovation rather than insurmountable barriers. Emerging research directions that combine stimuli-responsive materials with adaptive wedge geometries hold the potential to yield next-generation devices with environmental intelligence capabilities. These evolutionary developments not only validate the enduring relevance of bioinspired engineering principles, but also herald a new era of intelligent surface functional devices, with applications ranging from real-time pollution monitoring to point-of-care biomedical diagnostics.

## 6. Conclusions

This comprehensive review explores the transformative potential of wedge-shaped directional liquid transport (DLT) systems, synthesizing knowledge from biological prototypes, theoretical frameworks, fabrication advances, and emerging applications. Natural organisms, such as desert beetles, spider silk, cacti, and pitcher plants, serve as exemplary models of evolutionary optimization, where hierarchical structures leverage surface energy gradients, Laplace pressure differentials, and geometric confinement to facilitate energy-independent liquid transport. These biological principles have inspired the development of engineered systems, wherein wedge-shaped geometries synergistically integrate chemical wettability contrasts with curvature-driven forces, allowing for precise control over droplet dynamics. Theoretical analyses indicate that the interaction between surface energy gradients (as described by Young’s equation) and Laplace pressure gradients (resulting from asymmetric geometric confinement) forms the theoretical foundation for wedge-driven liquid transport. Recent advances in fabrication technologies, such as high-resolution 3D printing, laser micromachining, and dynamic masking, have successfully translated these principles into functional systems, overcoming previous challenges in scalability and precision. The applications of these systems are widespread, including water harvesting, condensation heat transfer, microfluidics, energy conversion, and wearable biosensing. These systems demonstrate superior efficiency in droplet collection, thermal management, and fluid manipulation when compared to conventional designs. However, despite these advances, several challenges remain. Performance degradation under real-world conditions, limited adaptability to dynamic environmental changes, and unresolved trade-offs between precision and scalability continue to impede the widespread adoption of DLT systems. Moreover, the integration of stimuli-responsive materials and machine learning-driven optimization techniques is still in its infancy, requiring further interdisciplinary collaboration to address these gaps. Future research should focus on three key strategic directions: (1) multi-physics integration, combining geometric, chemical, and thermal gradients to develop adaptive systems; (2) intelligent material systems, utilizing stimuli-responsive polymers and self-healing coatings to enhance environmental robustness; and (3) cross-scale manufacturing, connecting nanoscale interfacial engineering with macroscale structural design through hybrid techniques such as AI-optimized 3D printing. By deciphering nature’s inherent design principles and integrating them with computational modeling, next-generation DLT systems have the potential to revolutionize sustainable water management, portable diagnostics, and energy-efficient thermal systems. This convergence of biomimetics, materials science, and intelligent manufacturing signifies a paradigm shift toward autonomous, multifunctional fluidic technologies with profound societal impact.

## Figures and Tables

**Table 1:** The water harvesting efficiency on different wedge-shaped pattern surfaces.

**Method**	**Water Collection Efficiency**	**Refs**
FAS modification and UV lithography	2.11–2.78 g/(cm^2^·h)	[17]
Alkali-assisted oxidation	1150% improvement compared to PET	[41]
Laser processing	644% improvement at tilt angles of 30	[35]
Alkali-assisted oxidation	510% improvement compared to Cu(OH)_2_ surfaces	[55]
Spray-coating	14.9 ± 0.2 mg·min⁻^1^·cm⁻^2^	[46]
Spray-coating + laser-engraved	93.18 kg/m^2^·h	[32]
3D printing and laser scanning	4258.59 mg·cm⁻^2^·h⁻^1^	[57]
Alkali-assisted surface oxidation	7421 mg·h⁻^1^·cm⁻^2^	[37]
Spray-coating	14.9 ± 0.2 mg·min⁻^1^·cm⁻^2^	[47]
Laser-engraved	93.18 kg/m^2^·h	[58]
Alkali-assisted oxidation	7421 mg·h⁻^1^·cm⁻^2^	[49]
Laser ablation	5.6 kg·m⁻^2^·h⁻^1^	[59]

**Table 2:** The heat transfer coefficient improvement on different wedge-shaped pattern surfaces.

Method	Heat Transfer Coefficient	Refs
Laser-patterned masking + chemical etching	12.7% improvement compared to unpatterned surfaces	[5]
Chemical etching + boiling water passivation	34.4% enhancement compared to untreated aluminum	[7]
Laser ablation	Evaporation rate is 25% higher than the homogeneous surface	[24]
Laser ablation	30% improvement compared to pure superhydrophobic surfaces	[28]

## Data Availability

Not applicable.
